# Explaining and avoiding failure modes in goal-directed generation of small molecules

**DOI:** 10.1186/s13321-022-00601-y

**Published:** 2022-04-01

**Authors:** Maxime Langevin, Rodolphe Vuilleumier, Marc Bianciotto

**Affiliations:** 1 Molecular Design Sciences - Integrated Drug Discovery, Sanofi R&D, 94400 Vitry-sur-Seine, France; 2 PASTEUR, Département de chimie, École Normale Supérieure, PSL University, Sorbonne Université, CNRS, 75005 Paris, France

**Keywords:** De novo design, Generative models, Goal-directed generation, Recurrent neural network, Reinforcement learning, Failure modes, Quantitative structure-activity relationship, QSAR

## Abstract

Despite growing interest and success in automated in-silico molecular design, questions remain regarding the ability of goal-directed generation algorithms to perform unbiased exploration of novel chemical spaces. A specific phenomenon has recently been highlighted: goal-directed generation guided with machine learning models produce molecules with high scores according to the optimization model, but low scores according to control models, even when trained on the same data distribution and the same target. In this work, we show that this worrisome behavior is actually due to issues with the predictive models and not the goal-directed generation algorithms. We show that with appropriate predictive models, this issue can be resolved, and molecules generated have high scores according to both the optimization and the control models.

**Supplementary Information:**

The online version contains supplementary material available at 10.1186/s13321-022-00601-y.

## Introduction

Identifying a drug candidate is a long and complex task. It requires finding a compound that is active on the therapeutic target of interest, and that also satisfies multiple criteria related to safety and pharmacokinetics. To speed up this difficult search, de-novo drug design aims at finding promising novel chemical structures in-silico. The two principal use cases of generative de-novo drug design [1] are distribution learning, where the goal is to design new molecules that resemble an existing set of molecules, and goal-directed generation. The second use case is goal-directed generation [1]. In goal-directed generation, a generative model designs small molecules that maximize a given scoring function. The scoring function takes as input a molecular structure, and by the means of in-silico computations, returns a score that reflects the suitability of the molecular structure in a drug discovery setting. The scoring function is usually a combination of predicted biological and physico-chemical properties. Those predictions (widely refered to as QSPR models in the literature, for Quantitative Structure Property Relationships) are often computed by machine learning models [2–4]. While those models have shown impressive predictive accuracy on many drug-discovery related tasks [5], their performances deteriorate outside of their validity domains [6] and they can easily be fooled [7]. Novel molecules with high scores can be designed with deep generative models [2] coupled with reinforcement learning or other optimization techniques [8, 9]. Classical optimization methods, such as genetic algorithms [10, 11], have also shown good performance in goal-directed generation [1].

In a recent study published by Renz et al. [12], the authors identified failure modes of goal-directed generation guided by machine learning models. As they highlight in their work, optimization of molecules with respect to scoring functions can be performed in an unintended manner during goal-directed generation. Machine learning models are not oracles, and there are many reasons that can lead machine learning models to make erroneous predictions, such as distribution shift at test time [13], inherent limitations of the model or adversarial examples [14]. Furthermore, condensing every requirement of a drug-discovery project in a single score is not necessarily feasible. As the finality of goal-directed generation is to identify promising bioactive molecules for drug-discovery, identifying how and why goal-directed generation guided by machine learning can fail is of paramount importance for the adoption and success of those algorithms in drug-discovery.

### Experimental setup and results of the original study

**Fig. 1:**
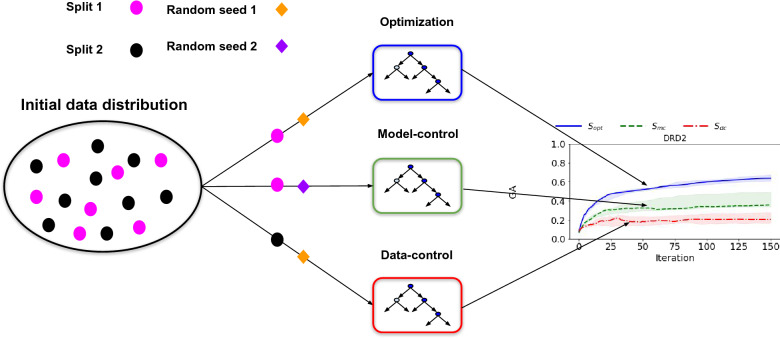
Experimental setup described by Renz and al. [12]. The initial dataset is split in two sets. The first split is used as a training set for the optimization model and the model-control model, and the second split for the data-control model. For a given molecule, the optimization (resp. model-control, data-control) score $S_{opt}$ (resp. $S_{mc}$, $S_{dc}$) is given by the optimization model’s (resp. model-control model’s, data-control model’s) predicted probability of being active The optimization score is used to guide goal-directed generation, and the evolution of control scores is also tracked during optimization. While the optimization score $S_{opt}$ grows throughout training, the control scores $S_{mc}$ and $S_{dc}$ stagnates and reaches much lower values

**Fig. 2:**
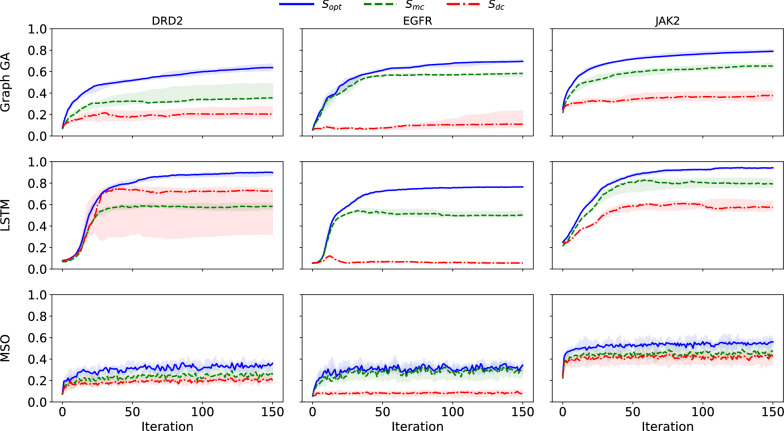
Reproduction of the results presented in Renz et al. trajectories of $S_{opt}$, $S_{mc}$, and $S_{dc}$, throughout the course of optimization on the three DRD2, EGFR and JAK2 datasets. Each of the three goal-directed generation algorithms (Graph GA, LSTM and MSO) were run 10 times. The line is the median of the means of scores for each run, and the envelope the $97.5 \%$ interval

In their study, Renz et al. design an experiment to assess whether goal-directed generation exploits features that are unique to the predictive model used for optimization, which is outlined in Fig. 1. Three datasets extracted from ChEMBL have been considered. Starting from a given dataset, they split it in two stratified random sets *Split 1/2*, where the ratio of actives to inactives is kept equal in both splits. On each of them, three bioactivity models are built in such a way that they should have a relatively equivalent predictive performance. The three classifiers are: a classifier $C_{opt}$ trained on *Split 1* (that takes as input a molecule *x* and returns the confidence score $S_{opt}(x)$, which is called the optimization score), another classifier $C_{mc}$ trained on the same split with a different random seed (that yields the model control score $S_{mc}$), and finally a classifier $C_{dc}$ trained on the *Split 2* (that yields the data control score $S_{dc}$). All three classifiers are Random Forests models [15] that share a similar architecture (see “Methods” section), and differ only by the random seed they are initialized with ($C_{opt}$ and $C_{mc}$) or the sample from the data distribution they are trained on ($C_{opt}$ and $C_{dc}$). These scores are comprised between 0 and 1, and correspond to the confidence score (given by the ratio of the number of trees predicting that a compound is active) returned by Random Forest classification models. $S_{opt}$ confidence score is used as a reward function for goal-directed generation that is performed with three different goal-directed generation algorithms (SMILES-based LSTM [3], Graph Genetic Algorithm [10] (Graph GA), and Multiple Swarm Optimization (MSO) [8]) (see “Methods” section for more details on the goal-directed generation algorithms). As the three bioactivity models are trained to predict the same property on the same data distribution, a practitioner would assume generated molecules with high $S_{opt}$ to also have high $S_{mc}$ and $S_{dc}$. Indeed, QSAR models built with different random seeds or train/test split are most of the time treated as interchangeable by practitioners. In a highly valuable critical analysis, Renz et al. highlighted several issues related to distribution learning and goal-directed generation. For the latter, they observe that while $S_{opt}$ grows during goal-directed generation, $S_{mc}$ and $S_{dc}$ diverge from the optimization score during the course of the optimization, reaching on average lower values than $S_{opt}$ (see Fig. 2 and “Methods” section for further details) and sometimes even decrease throughout the course of optimization. Those results, suggesting that the molecules produced through goal-directed generation exploit bias unique to the model they are optimized on, were noted in the literature [16, 17]. Indeed, those results are concerning as they cast doubt on the viability of generating optimized molecules guided by machine learning models.

To avoid the pitfall of designing molecules with high optimization scores and low control scores, Renz et al. suggest to stop goal-directed generation when control scores stop increasing. This requires to hold out a significant part of the original dataset to build a data control model: this might not be feasible in low data regimes, and would harm the predictive power of the optimization models used during goal-directed generation.

### Interpretation of the initial results

The observed difference between $S_{opt}$, $S_{mc}$ and $S_{dc}$ is explained in [12] by the fact that goal-directed generation algorithms exploit bias in the scoring function, defined as the presence of features that yield a high $S_{opt}$ but do not generalize to $S_{mc}$ and $S_{dc}$ . The first case (high $S_{opt}$ and low $S_{mc}$) is referred to as exploiting model-specific biases. Indeed, $S_{opt}$ and $S_{mc}$ are scores given by two classifiers trained on the same data (only the model differs, through the choice of a different random seed). A molecule with a high $S_{opt}$ and low $S_{mc}$ therefore must exploit features that are specific to the exact model $C_{opt}$ and not shared by $C_{mc}$, even though those models were trained on the same data. Conversely, the second case (high $S_{opt}$ and low $S_{dc}$) is referred to as exploiting data specific biases. The authors interpret this as a failure of the goal-directed generation procedure: “there is a mismatch between optimization scores and data control scores, which shows that the optimization procedure suffers from model and/or data specific biases”. This interpretation is further supported by the fact that the difference between optimization and control scores grows over time during goal-directed generation.

Formally, we can view a dataset of molecules and associated bioactivities as a sample $(X_i, y_i), i \in [\![1;n]\!] \sim P$, where $(X_i, y_i)$ denote a molecule and its associated bioactivity, and *P* is the distribution of the dataset. Most classifiers return a decision function or a confidence score $S_{classifier}(x)$, that can be used as scoring functions when searching for novel active compounds using goal-directed generation algorithms. This is achieved by maximizing $S_{classifier}(x)$, through optimization techniques or reinforcement learning. As the three classifiers $C_{opt}$, $C_{mc}$ and $C_{dc}$ model the same property on the same dataset, Renz et al. expect molecules obtained with goal-directed generation to be also predicted active by the classifiers $C_{mc}$ and $C_{dc}$, and to have similar control scores as their optimization scores. As $S_{mc}$ and $S_{dc}$ are significantly lower than $S_{opt}$, the conclusion reached is that the molecules generated are predicted active by $C_{opt}$ for the wrong reasons (the exploitation of biases, a behavior already observed in the machine learning literature [18]) as those predictions do not translate in similar bioactivity models. The intuition behind this conclusion is that features that are true explanatory factors of the output will yield high scores both by the optimization and control models. Therefore, a goal-directed generation algorithm that design molecules with high optimization scores but low control scores could be exploiting spurious features specific only to the optimization model, that will not translate when testing the molecule in a wet-lab experiment [12].

This conclusion rests on the unproven assumption that, in the original data distribution *P*, molecules predicted active with high confidence by the optimization model $C_{opt}$ are also predicted active with high confidence by the control models. This hypothesis might seem reasonable considering that all three models share the same architecture and are trained to predict the same property on the same data distribution [12]. However, the goal of this work is precisely to test this assumption. Modeling biological properties from chemical structure is a difficult task, especially on the small, noisy and chemically very diverse (see “Methods” section) datasets used in [12]. While the optimization and control models display similar predictive performance metrics (as assessed by the ROC-AUC metric), it does not imply that $S_{opt}$ will perfectly correlate with control scores $S_{mc}$ and $S_{dc}$. It is therefore necessary to validate this assumption in order to assign the failure of goal-directed generation to the goal-directed generation algorithms themselves, and not to the initial difference observed on the data distribution. This requires a comparison of $S_{opt}$ with $S_{mc}$ and $S_{dc}$ on an independent sample of molecules from the initial data distribution *P*.

In this work, we show that the difference observed between optimization and control scores on goal-directed generation tasks is not due to a failure in the procedure itself, but that it can be explained by an initial difference between the scores of the classifiers on the original data distribution. We further show that the optimized population, in feature space, has indeed similar statistics as the original dataset, and that the divergence between optimization and control scores is already present in the initial dataset. We adapt the initial experimental setting by Renz et al. [12], in order to have an experimental setting that allows us to answer the question of whether goal-directed generation algorithm exploit model or data specific biases during optimization. We assess in those adequate settings whether we still observe the difference between optimization and control scores, and show that in those appropriate settings, the failure of goal-directed generation algorithms is not observed. Finally, we highlight that the behavior described in [12] warrants caution when designing predictive models for goal-directed molecular generation.

## Results

### Analyzing goal-directed generation failure modes

**Fig. 3:**
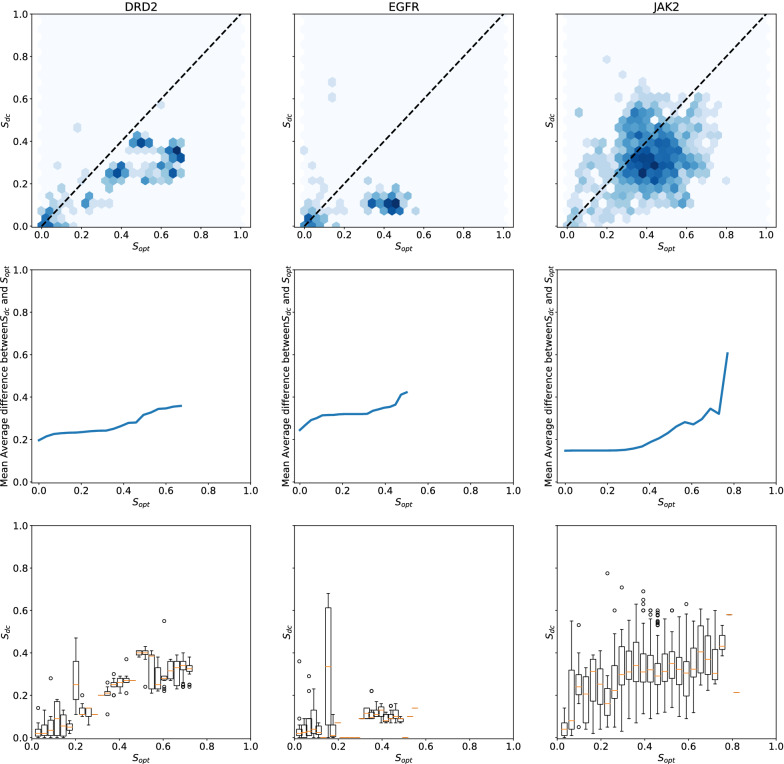
$S_{opt}$ and $S_{dc}$ in the EGFR, JAK2 and DRD2 Topliss-augmented datasets. From top to bottom: hexbin plots (log scale) of data control as a function of optimization score; Mean Average Difference between $S_{dc}$ and $S_{opt}$ as a function of $S_{opt}$ (at absciss *x*, the MAD plotted is the MAD for molecules with optimization scores higher than *x*); distribution of $S_{dc}$ (95 CI) as a function of optimization score. For the second and third row, the lines and boxplots stop at absciss $x_{max}$ for which there is no more samples with optimization scores higher than $x_{max}$

To assess whether molecules from the original distribution with high optimization score $S_{opt}$ also have high control scores $S_{mc}$ and $S_{dc}$, we need to have an independent sample from *P* that is used neither to build the optimization or the control models. We therefore start by splitting the dataset in a 90/10 fashion to obtain an held-out test set, and then proceed to split the 90$\%$ remaining of the dataset as described earlier to build $C_{opt}$, $C_{mc}$ and $C_{dc}$. We can then compare the values of $S_{opt}$, $S_{mc}$ and $S_{dc}$ on the molecules of the held-out test set. To obtain smoother estimates for the statistics we use, the held-out set is ten-fold augmented with analogs obtained through enumeration of Topliss trees, a classic medicinal chemistry exploration method (see “Methods” section for more details). Indeed, without data augmentation (see Additional file 1: Figure S9), the low number of molecules with high values of $S_{opt}$ would make the estimation of quantities of interest noisier than when using data augmentation (see Fig. 3). These results are displayed in Fig. 3 ($S_{opt}$ vs $S_{dc}$) and in Additional file 1: Figure S7 ($S_{opt}$ vs $S_{mc}$).

Analyzing how control scores evolve in function of optimization scores in the held-out set shows that there is already in the initial dataset a significant difference between optimization and control scores. When looking at the Mean Average Difference (MAD) between $S_{opt}$ and $S_{dc}$, we can see that this difference grows with $S_{opt}$, while it is not the case between $S_{opt}$ and $S_{mc}$. On the three datasets, the MAD reaches approximately a value of 0.3 on the molecules with the highest optimization scores. The fact that there is such a large difference between $S_{opt}$, $S_{mc}$ and $S_{dc}$ on the original data distribution challenges the conclusion from Renz et al. that the divergence observed during goal-directed generation is necessarily caused by a failure of goal-directed generation algorithms. To determine whether exploitation of model or data specific biases actually plays a role, we need to assess if the difference between the scores of classifiers observed on the initial dataset fully explains the observation made during goal-directed generation.

For instance, on the EGFR dataset, even the molecules with the highest $S_{opt}$ (between 0.5 and 0.6) have a $S_{dc}$ below 0.2. Without exploiting any bias and simply by maximizing $S_{opt}$ while staying in the original data distribution, we should not expect data control scores to reach values much above 0.2. Let us consider the distribution of optimization scores of sampled molecules at time step *t* by a goal-directed generation algorithm $P_{t}[S_{opt}(x)]$. From our held-out validation set, we can reliably approximate $P[S_{dc}(x)|S_{opt}(x)]$ and $P[S_{mc}(x)|S_{opt}(x)]$, the distribution of data control and model control scores observed on the initial dataset, conditioned on the value of the optimization score. At any time step *t*, the expected distribution of model control score is $P[S_{mc}(x)|S_{opt}(x)]P_{t}[S_{opt}(x)]$, and of data control score is $P[S_{dc}(x)|S_{opt}(x)]P_{t}[S_{opt}(x)]$. As we can sample from those distributions, this allows us to estimate an empirical tolerance interval (i.e., a statistical interval where at least a pre-specified percentage of a population is expected to fall, see “Methods” section for more details) for data and model control scores at each time step. We compute those tolerance intervals for expected model and data control scores along the trajectories of goal-directed generation algorithm (see “Methods” section for more details). As long as the data or model control scores observed during goal-directed generation fall above the lower bound of the tolerance intervals, we can conclude that the difference between $S_{opt}$, $S_{mc}$ and $S_{dc}$ can be explained independently of an hypothetical failure of the goal-directed generation algorithms.

### Implications

**Fig. 4:**
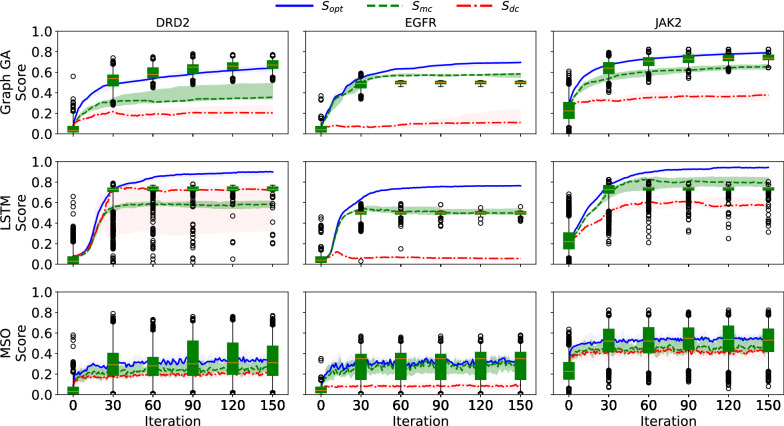
Trajectories of $S_{opt}$, $S_{mc}$ and $S_{dc}$ superimposed with tolerance intervals for $S_{mc}$. For $S_{opt}$, $S_{mc}$ and $S_{dc}$, the mean of the scores of each run are computed. The bold line corresponds to the median over those means while the shaded areas correspond to the interquartile range [12]. Here, the shaded area that is the more visible (green) corresponds to the interquartile range of the model control scores. The full range of tolerance intervals for the expected model control scores are shown every 30 iterations

**Fig. 5:**
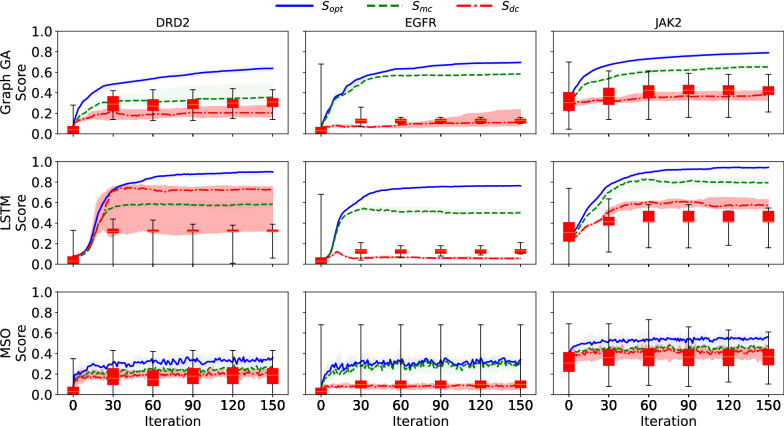
Trajectories of $S_{opt}$, $S_{mc}$ and $S_{dc}$ superimposed with tolerance intervals for $S_{dc}$. Here, the shaded area that is the more visible (red) corresponds to the interquartile range of the data control scores. The full range of tolerance intervals for the expected data control scores are shown every 30 iterations

**Fig. 6:**
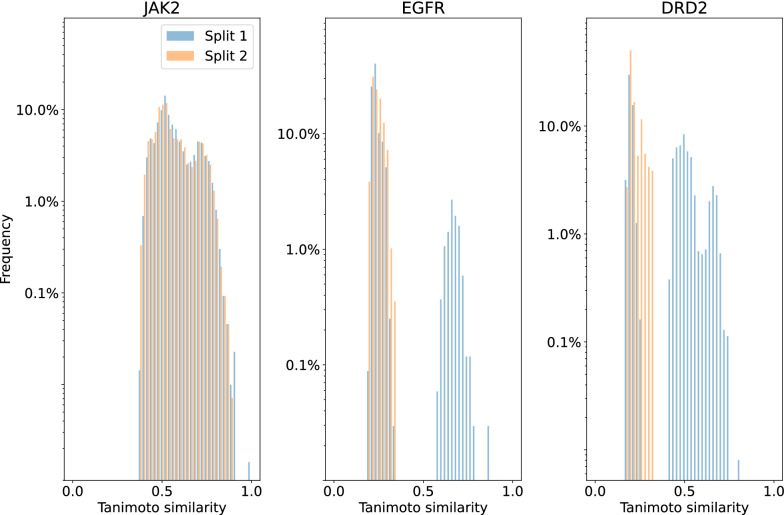
Similarities to *Split 1* and *2* of the top-5 $\%$ scored molecules (optimization score) in an external test set, showing pre-existing bias in the original distribution. y-axis is in logarithmic scale. Similarity between molecules is measured by the Tanimoto similarity between Morgan fingerprints of radius 2 and 1024 bits, computed with the RDKit

As shown in Figs. 4 and 5, the trajectories of control scores during goal-directed generation do not fall below the tolerance interval of the adjusted scores. This shows that we cannot rule out that the observed difference between optimization and control scores [12] is fully explained by the difference between classifiers on the original data distribution. Furthermore, besides the difference between optimization and control scores, a bias towards generating molecules similar to *Split 1* was also observed in the original study [12]. This was analyzed as a result of data-specific bias, and was another argument in favor of the failure of goal-directed generation algorithms. We assessed whether the bias observed (i.e., molecules sampled during goal-directed generation being more similar to those of *Split 1* than *Split 2*) was already present in the initial dataset. The top-5 percentile (with regard to $S_{opt}$) of molecules from the held-out set were selected, and their similarities with molecules from *Split 1* and *2* were computed (see Fig. 6). For the EGFR and DRD2 datasets, a large bias towards molecules from *Split 1* was observed. As for the difference between optimization and control scores, the biases observed in molecules sampled during goal-directed generation were already present in the initial dataset. It is therefore possible that the underlying problem is not caused by goal-directed generation algorithm but rather by the classifiers themselves, that do not correlate on an independent sample of the initial dataset.

Our results show that initial differences between control and optimization scores, as well as the biases towards molecules that built the optimization model, explain by itself the behavior observed by Renz et al. Nonetheless, it does not prove the converse statement. It is indeed still possible that goal-directed generation algorithms do exploit biases, and questions remain around the validity of the output of goal-directed generation algorithms, as molecules sampled tend to be unstable, unsynthesizable or have highly uncommon fragments [12]. It should also be noted that the biases observed on the initial data distribution might be due to the datasets used in Renz et al. which are extracted from public data on three bioactivity assays (EGFR, JAK2 and DRD2) and suffer from shortcomings that might hamper QSAR modeling of bioactivities: they are rather small (842, 667, and 842 molecules, respectively), with a low number of active molecules (40, 140, and 59 active molecules), and are heterogeneous. For example, see Additional file 1: Table S1 for a set of compounds in the DRD2 actives whose activity or mode of action are questionable.

### Building appropriate experimental setups

As highlighted in the previous section, there was an initial disagreement between optimization and control scores on the datasets used in the original study on goal-directed generation failure modes, as well as a bias towards *Split 1* in molecules with high $S_{opt}$. This explains, as shown in Figs. 4, 5 and 6, the problematic behavior described by Renz et al. in their original study on failure modes of goal-directed generation algorithms. Does this behavior still appear when optimization and control scores correlate well on the initial data distribution? To tackle this question, we build two tasks where the difference between optimization and control scores on the initial data distribution is much lower than in the three tasks discussed earlier, to check whether goal-directed generation algorithm actually exploits specific biases that lead to high optimization scores $S_{opt}$ but low control scores $S_{mc}$ and $S_{dc}$.

**Fig. 7:**
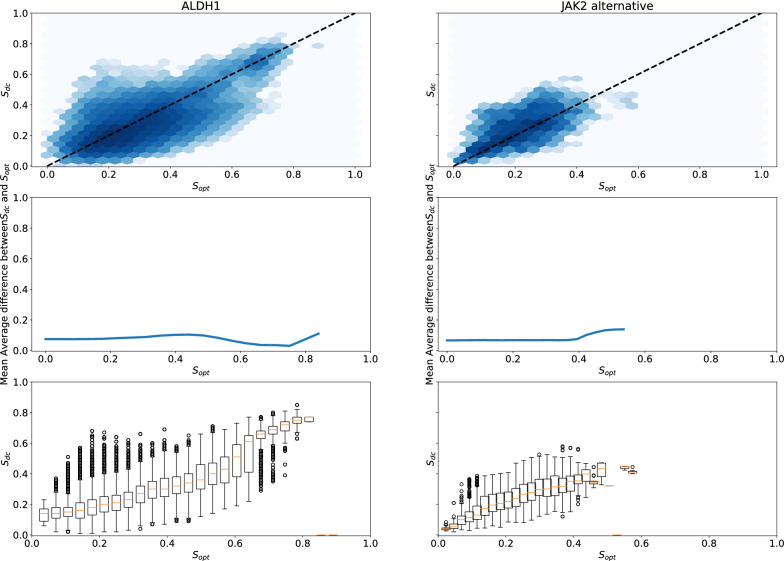
$S_{opt}$, $S_{mc}$ and $S_{dc}$ for the ALDH1 and the JAK2 datasets (with modified predictive model architecture). From top to bottom: hexbin plots (log scale) of data control as a function of optimization score; Mean Average Difference between $S_{dc}$ and $S_{opt}$ as a function of $S_{opt}$ (at absciss *x*, the MAD plotted is the MAD for molecules with optimization scores higher than *x*); distribution of $S_{dc}$ (95 CI) as a function of optimization score. For the second and third row, the lines and boxplots stop at absciss $x_{max}$ for which there is no more samples with optimization scores higher than $x_{max}$

The first task relies on a public dataset of molecules and associated bioactivities on the ALDH1 target. This dataset, with 464 molecules, is extracted from the Lit-PCBA database [19]. In order to build two similar splits, after a held out set was randomly selected, molecules were paired as to maximize intra-pair similarities (see “Methods” section for more details) and a molecule for each pair was assigned to either *Split 1* or *Split 2*. For the second task, we rely on the JAK2 dataset already used in the original study by Renz et al. We kept the JAK2 dataset as it was homogeneous, with molecules that seem reasonable from a medicinal chemistry point of view. To construct classifiers that agree on the initial data distribution, we change modeling choices by augmenting the minimum number of samples per leaf and augmenting the number of trees of the random forests. Those choices tend to avoid exploiting spurious correlations that would lead to disagreement between the different classifiers on the initial data distribution. In those two tasks, there is very limited difference between $S_{opt}$, $S_{mc}$ and $S_{dc}$ on a held-out test set, as indicated in Fig. 7.

This makes those use cases good experimental settings to test whether goal-directed generation algorithm actually exploit biases that leads to the generation of molecules with high optimization scores but low control scores.

### Results on appropriate datasets

On the two cases described in the previous section, we run the three same goal-directed generation algorithms as Renz et al. The only difference with our procedure is that we used the test set as our starting population (as pretraining for the SMILES-LSTM and starting population for Graph GA and MSO). If those algorithms behave as intended, we shouldn’t see major differences between optimization and control scores during the course of optimization.

**Fig. 8:**
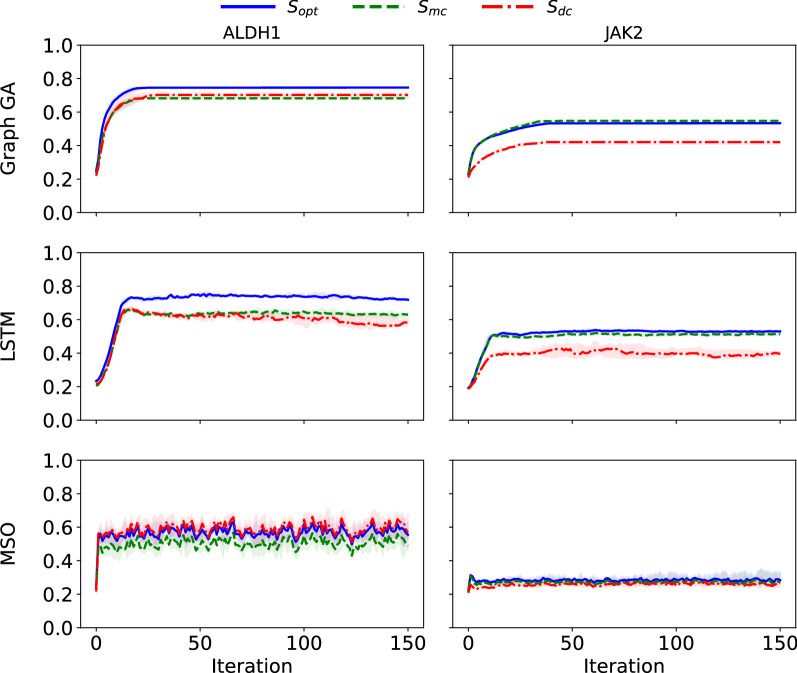
Median optimization and control scores on the ALDH1 and the JAK2 (with modified architecture for the predictive model) datasets

As shown in Fig. 8, in our experimental setup, there is no major difference between control and optimization scores, and the difference observed in Renz et al. seems to be in a large part due to initial difference between optimization and control scores on the original data distribution.

## Discussion

Our analysis and experiments around failure mechanisms of goal-directed generation algorithms allows us to reinterpret the results observed previously [12]. We show that the explanation of the difference in optimization and control scores rests not in the exploitation of biases specific to the optimization model, but in a difference between optimization and control scores on the initial data distribution. When no major difference between optimization and control scores is observed on the initial distribution, and simple conditions (especially regarding the starting population for the goal-directed generation algorithms) are respected, the problematic behavior observed by Renz et al. disappears. Nevertheless, on small and noisy datasets, many different predictive models can be built with similar predictive performance, and yet disagree on the highest-scoring molecules. This might be specific to classification models, and a study of this behavior for regression models would be a further avenue of research. The problem can be reformulated: if several models can be built with no way to discriminate between them (e.g., they have roughly the same validation metrics such as AUC on a validation set), and if those models attribute high scores to different molecules, then the choice of one particular model for optimization will bias goal-directed generation algorithms towards the specific molecules that are highly scored by the optimization model. As highlighted in our work, this problem is due to a limited ability of predictive models to extract signal from data, rather than to a problem from the goal-directed generation algorithms themselves. It nevertheless shows that caution should prevail when selecting a predictive model for goal-directed generation, as under some conditions, the choice of a particular predictive model might bias goal-directed generation. While our study and the one by Renz et al. focused on QSAR models built with ECFP descriptors, this problematic behavior is also seen with other molecular descriptors such as physico-chemical descriptors or Atom-Pair fingerprints (see Additional file 1: Figures S10 and S11).

The fact that this failure mode of goal-directed generation is not observed (or rather in a much more limited way) when optimization and control models agree on the initial distribution is rather reassuring. This adequate behavior is nonetheless dependent on selecting an adequate initial population, and on agreement of different models on the initial distribution. As we have shown, this can be influenced either by the dataset itself, or the type and architecture of the machine learning model used. As shown in Additional file 1: Figure S10 on the DRD2 dataset, an adequate choice of descriptors can also mitigate the disagreement between optimization and control models.

Designing novel molecules that have high scores according to control models is an argument in favor of goal-directed generation algorithms behaving as expected. Nonetheless, it is important to keep in mind that the primary goal of goal-directed generation is to generate useful molecules for drug-discovery projects. This implies that the molecules produced should behave well in feature space (e.g., have good scores according to various predictive models for the same property), but should also avoid other pitfalls observed in goal-directed generation such as poor synthesizability, unexpected fragments or being highly reactive [12, 20] (see also Additional file 1: Figures S2 and S5 for low-quality structures generated on the ALDH1 and JAK2 datasets by the RNN-LSTM algorithm). Our work shows that the two problems identified by Renz et al. namely the difference between $S_{opt}$, $S_{mc}$ and $S_{dc}$, and the poor quality of generated structures, are rather independent. Indeed, even in tasks where control scores follow the optimization score, the molecules generated can be irrelevant from a drug-discovery perspective. Evaluating ways to constrain goal-directed generation to focus on exploring relevant chemical space only is therefore a still open and important question for applications of generative models to drug design. Studying how goal-directed generation algorithms behave when coupled with machine learning that operate directly on molecular graphs [21] would also be of interest. Indeed, for such models the feature space is directly the space of molecular graphs. A goal-directed generation algorithm that behaves as intended in feature space should thus produce satisfying molecules as well.

## Conclusions

To summarize, we show that failure modes of goal-directed generation algorithms observed in Renz et al. [12] can be explained by a disagreement between predictive models on the original data distribution. This is probably due to small, noisy datasets where predictive models used are underspecified. Together, these results highlight the importance of the quality of datasets and predictive models for goal-directed generation. We show how to detect settings where this behavior might arise, and describe how to design adequate datasets and model specifications, such as to avoid this problematic behavior.

While our work is reassuring on the ability of goal-directed generation to perform the task at hand, it should be noted that other problems, described by Renz et al. are present. Especially, goal-directed generation can be oblivious to common medicinal chemistry knowledge, and finding satisfying molecules in the feature space used for predictive modeling is different from finding satisfying molecules for a drug-discovery project.

An opportunity for future research will be to understand why goal-directed generation algorithms, despite being able to design novel molecules that score well even according to control models, still produce low-quality chemistry that slows down further adoption of those technologies in drug discovery projects.

## Methods

### Datasets

The three datasets from the original study by Renz et al. were extracted from ChEMBL. For the JAK2 dataset, compounds with a pIC50 greater than 8 were labeled as actives, and for the EGFR and DRD2 datasets the labels were extracted from the “Comment” column (more details can be found in the original manuscript). The Aldehyde Dehydrogenase 1 (ALDH1) dataset was extracted from the LIT-PCBA [19] database. The LIT-PCBA database is a curated version of 149 dose-response PubChem [22] bioassays. The compounds were processed to remove false positives and assay artifacts, and to keep actives and inactives within similar range of molecular properties. The active compounds were clustered using RDKit [23] (with Morgan fingerprints of radius 2 and 2048 bits, LeaderPicker diversity picking, and a Tanimoto distance of 0.6) and the 6 most populated clusters were selected, which contain 18–32 actives. For each of the clusters, the inactive compounds closer than 0.6 (Tanimoto similarity) were also selected, leading to a total of 464 compounds. Among those, the 173 that contain the purine substructure were selected. 18 pairs of active and 46 pairs of inactive molecules were built by decreasing graph edit distance of their molecular graph (provided it was less than 10) using the networkx python library [24]. The 45 remaining molecules were used to evaluate the performance of the models generated using different splits between the pairs of molecules.

### Predictive models

The predictive models used are random forest classifiers [15], implemented in the python library scikit-learn [25]. The scoring function is given by the predicted probability of being active, which is the ratio of trees predicting that a compound is active. The features used are folded ECFP fingerprints [26], of size 1024 and radius 2. The RDKit [23] implementation was used (see the original manuscript by Renz et al. for more details). The parameters for the random forest classifiers were the scikit-learn default parameters, except for the modified JAK2 experiment, where the number of trees was set to 200 (instead of 100), and the minimum number of samples per leaf was set to 3 (instead of 1). In order to build the expected tolerance intervals (see main text) for $S_{mc}$ and $S_{dc}$, we had to fix the random seeds that parameterize the different predictive models. This is different from what was done in the original manuscript, and could explain the slight discrepancy observed when reproducing their results, as seen in Fig. 2.

### Goal-directed generation algorithms

The three goal-directed generation algorithms used are the same as in the original study by Renz et al. The first one is a genetic algorithm that operates on molecular graphs [10]. This algorithm starts with an initial population, that is updated through series of mutations (changing parts of a given molecular graph) and crossovers (combining two different graphs together). The highest scoring molecules are kept, enriching the population towards higher scores. The second one is a SMILES-based Long Short Term Memory network (LSTM), that is optimized through a hill-climbing algorithm [1]. The hill-climbing algorithm fine-tunes the LSTM on the best molecules that the same network generated at the previous time step. Finally, the third algorithm used is Multiple Swarm Optimization (MSO) [8] in the latent space encoded by the CDDD algorithm [27]. The MSO algorithm searches through the latent space with multiple particles that share common information. The two first algorithms were chosen because they displayed the best performance in the Guacamol benchmark [1], while the third algorithm was chosen as it is more representative of a large number of molecular optimization methods that rely on latent space optimization. The MSO and graph genetic algorithm are given a starting population, while the LSTM can be pretrained on a given population. In the work of Renz et al. those populations are a random subset of ChEMBL [28]. In this work, we use the held-out test set as a starting or pretraining population. Indeed, the algorithms should be initialized on the same population that the predictive models where trained on. Otherwise, a distribution shift is built in the procedure, which might lead to unsatisfying results.

### Topliss augmentation

Given that the number of compounds in the held out test set is low, we augment this set with structural analogs to obtain smoother estimates for the statistics we use when computing tolerance intervals. The goal of the held-out test set is simply to compare optimization and control scores on the initial data distribution. Therefore, we do not need the true labels of the molecules and can rely on data augmentation. The Topliss scheme [29] is a way to explore substituents on a phenyl ring in a drug design context. To augment the test set, we iterate over molecules in the set, and explore every phenyl ring of the molecule using the Topliss scheme, providing many structural analogs to the initial molecule. Those structural analogs are by definition reasonable from a medicinal chemistry point of view.

### Comparing the evolution of optimization and control scores throughout optimization

The three goal-directed generation algorithms are run for 151 epochs. For each task, 10 different runs of each algorithms is performed. For each run, the mean of the scores at each time step is kept, and the distribution of the means (95 CI, median in bold) is shown.

### Tolerance intervals for control scores

To get tolerance intervals for expected data control scores, the scores at each time step are treated as sample from a probability distribution $P_{t}[S_{opt}(x)]$. The range of optimization score is divided into 25 equal parts, for which empirical distribution of control scores are known (see bottom rows of Figs. 3 and 7). For each sample from $P_{t}[S_{opt}(x)]$ (i.e., for each score), 10 samples of $P[S_{dc}(x)|S_{opt}(x)]$ and $P[S_{mc}(x)|S_{opt}(x)]$ are drawn. This procedure allows to sample from the expected control scores, and to derive empirical tolerance interval. Tolerance interval at $\alpha \%$ are defined as intervals where $\alpha \%$ of a given population will lie. Here, tolerance intervals at $95 \%$ are shown. If the actual control scores lie within those tolerance intervals, then the difference between control and optimization scores can be explained by the difference between control and optimization scores on the initial data distributions. The disagreement between $S_{opt}$, $S_{mc}$ and $S_{dc}$ highly depends on the value of $S_{opt}$. Therefore, we do not report measures such as Pearson’s coefficient of correlation $R^2$, as it gives less information on the local disagreement between optimization and control scores than the reported Mean Average Difference.

## Supplementary Information

**Additional file 1:** **Table S1.** Selection of questionable active compounds from the DRD2 dataset. **Figure S1.** Random set of the final samples generated on the ALDH1 dataset with the graph genetic algorithm. **Figure S2.** Random set of the final samples generated on the ALDH1 dataset with the SMILES-LSTM algorithm. Those chemical structures are highly non drug-like due to e.g., the repetition of tetrazole rings, long chains with sulphur-sulphur bonds and other heteroaromatic-heteroaromatic single bonds. **Figure S3.** Random set of the final samples generated on the ALDH1 dataset with the MSO algorithm. **Figure S4.** Random set of the final samples generated on the JAK2 (with modified architecture for the predictive model) dataset with the graph genetic algorithm. **Figure S5.** Random set of the final samples generated on the JAK2 (with modified architecture for the predictive model) dataset with the SMILES-LSTM algorithm. We observed similar non drug-like patterns as in Additional file 1: Figure S2. **Figure S6.** Random set of the final samples generated on the JAK2 (with modified architecture for the predictive model) dataset with the MSO algorithm. **Figure S7.** *S*_*opt*_ and *S*_*mc*_ in the DRD2, EGFR and JAK2 Topliss-augmented datasets. From top to bottom: hexbin plots (log scale) of data control as a function of optimization score; Mean Average Difference between *S*_*mc*_ and *S*_*opt*_ as a function of Sopt (at absciss *x*, the MAD plotted is the MAD for molecules with optimization scores higher than *x*); distribution of Smc (95 CI) as a function of optimization score. For the second and third row, the lines and boxplots stop at absciss xmax for which there is no more samples with optimization scores higher than *x*_*max*_. **Figure S8.** *S*_*opt*_ and *S*_*mc*_ in the ALDH1 and JAK2 modified Topliss-augmented datasets. From top to bottom: hexbin plots (log scale) of data control as a function of optimization score; Mean Average Difference between *S*_*mc*_ and *S*_*opt*_ as a function of *S*_*opt*_ (at absciss *x*, the MAD plotted is the MAD for molecules with optimization scores higher than *x*); distribution of *S*_*dc*_ (95 CI) as a function of optimization score. For the second and third row, the lines and boxplots stop at absciss *x*_*max*_ for which there is no more samples with optimization scores higher than *x*_*max*_. **Figure S9.** *S*_*opt*_ and *S*_*dc*_ in the DRD2, EGFR and JAK2 datasets without Topliss augmentation. The fact that only a few molecules have a high *S*_*opt*_ makes the evaluation of related quantities noisier than with data augmentation. From top to bottom: hexbin plots (log scale) of data control as a function of optimization score; Mean Average Difference between *S*_*dc*_ and *S*_*opt*_ as a function of Sopt (at absciss *x*, the MAD plotted is the MAD for molecules with optimization scores higher than *x*); distribution of Smc (95 CI) as a function of optimization score. For the second and third row, the lines and boxplots stop at absciss *x*_*max*_ for which there is no more samples with optimization scores higher than *x*_*max*_. **Figure S10.** *S*_*opt*_ and *S*_*dc*_ in the DRD2 (ROC-AUC: 0.78, Average Precision: 0.2), EGFR (ROC-AUC:0.93, Average Precision: 0.67) and JAK2 (ROC-AUC:0.69, Average Precision: 0.41) Topliss-augmented datasets, when the classifiers use a combination of physico-chemical descriptors (number of hydrogen bonds donors and acceptors, number of rings, number of rotatable bonds, total polar surface area, Crippen descriptors (ClogP and molar refractivity), molecular weight, fraction of SP3 carbons, the ratio of atoms in the Murcko scaffold on the total number of heavy atoms, the number of heavy atoms, the maximum and minimum cycle size, the minimal, maximal and total charge and the number of chiral centers, as implemented in the RDKit). From top to bottom: hexbin plots (log scale) of data control as a function of optimization score; Mean Average Difference between *S*_*dc*_ and *S*_*opt*_ as a function of *S*_*opt*_ (at absciss *x*, the MAD plotted is the MAD for molecules with optimization scores higher than *x*); distribution of Smc (95 CI) as a function of optimization score. For the second and third row, the lines and boxplots stop at absciss xmax for which there is no more samples with optimization scores higher than xmax. **Figure S11.** *S*_*opt*_ and *S*_*dc*_ in the DRD2 (ROC-AUC: 0.95, Average Precision: 0.56), EGFR (ROC-AUC:0.88, Average Precision: 0.24) and JAK2 (ROC-AUC:0.67, Average Precision: 0.37) Topliss-augmented datasets, when the classifiers use Atom-Pair descriptors (as implemented in the RDKit). From top to bottom: hexbin plots (log scale) of data control as a function of optimization score; Mean Average Difference between Sdc and Sopt as a function of Sopt (at absciss *x*, the MAD plotted is the MAD for molecules with optimization scores higher than *x*); distribution of Smc (95 CI) as a function of optimization score. For the second and third row, the lines and boxplots stop at absciss *x*_*max*_ for which there is no more samples with optimization scores higher than *x*_*max*_.

## Data Availability

The code and datasets supporting the conclusions of this article are available at https://github.com/Sanofi-Public/IDD-papers-avoiding_failure_modes.
