# Supplementary material for: Explaining and avoiding failure modes in goal-directed generation of small molecules
Source: J Cheminform. 2022 Apr 1;14:20. doi: 10.1186/s13321-022-00601-y (PMC8973583; doi:10.1186/s13321-022-00601-y)
Supplement: Supplementary file 1 — Additional file 1: Table S1. Selection of questionable active compounds from the DRD2 dataset. Figure S1. Random set of the final samples generated on the ALDH1 dataset with the graph genetic algorithm. Figure S2. Random set of the final samples generated on the ALDH1 dataset with the SMILES-LSTM algorithm. Those chemical structures are highly non drug-like due to e.g., the repetition of tetrazole rings, long chains with sulphur-sulphur bonds and other heteroaromatic-heteroaromatic single bonds. Figure S3. Random set of the final samples generated on the ALDH1 dataset with the MSO algorithm. Figure S4. Random set of the final samples generated on the JAK2 (with modified architecture for the predictive model) dataset with the graph genetic algorithm. Figure S5. Random set of the final samples generated on the JAK2 (with modified architecture for the predictive model) dataset with the SMILES-LSTM algorithm. We observed similar non drug-like patterns as in Additional file 1: Figure S2. Figure S6. Random set of the final samples generated on the JAK2 (with modified architecture for the predictive model) dataset with the MSO algorithm. Figure S7. Sopt and Smc in the DRD2, EGFR and JAK2 Topliss-augmented datasets. From top to bottom: hexbin plots (log scale) of data control as a function of optimization score; Mean Average Difference between Smc and Sopt as a function of Sopt (at absciss x, the MAD plotted is the MAD for molecules with optimization scores higher than x); distribution of Smc (95 CI) as a function of optimization score. For the second and third row, the lines and boxplots stop at absciss xmax for which there is no more samples with optimization scores higher than xmax. Figure S8. Sopt and Smc in the ALDH1 and JAK2 modified Topliss-augmented datasets. From top to bottom: hexbin plots (log scale) of data control as a function of optimization score; Mean Average Difference between Smc and Sopt as a function of Sopt (at absciss x, the MAD plotted is the MAD for [file 13321_2022_601_MOESM1_ESM.pdf]

Supplementary Information

Explaining and avoiding failure modes in goal-directed generation of small molecules

Maxime Langevin[1,2]
, Rodolphe Vuilleumier[2]
and Marc Bianciotto[1*]

*Correspondence:
marc.bianciotto@sanofi.com
[1]Molecular Design Sciences - Integrated Drug Discovery, Sanofi RD, 94400 Vitry-sur-Seine, France
Full list of author information is available at the end of the article

Samples from the DRD2 dataset

Table S1 shows several examples of questionable active compounds retrieved from the DRD2 dataset. Those samples suggest that the detected activities on the assay (radioactive ligand binding competition assay) might come from different mechanisms. This heterogeneity, coupled with the small size of the dataset, could explain the observed difference between optimization and control scores.

Samples generated on the ALDH1 dataset

Figure S1, Figure S2 and Figure S3 display final samples generated by the three goal-directed algorithms. Even when optimization and control scores do not diverge, issues concerning the quality of molecules generated remain.

Samples generated on the JAK2 dataset with modified predictive model

Figure S4, Figure S5 and Figure S6 display final samples generated by the three goal-directed algorithms. Even when optimization and control scores do not diverge, issues concerning the quality of molecules generated remain.

Model-control score and optimization score comparison.

Author details
[1]Molecular Design Sciences - Integrated Drug Discovery, Sanofi RD, 94400 Vitry-sur-Seine, France. [2]PASTEUR, Département de chimie, École Normale Supérieure, PSL University, Sorbonne Université, CNRS, 75005 Paris, France.

References

1. Tredici, A.L.D., Ma, J.-N., Piu, F., Burstein, E.S.: Identification of the antiarrhythmic drugs amiodarone and lorcainide as potent h3 histamine receptor inverse agonists. Journal of Pharmacology and Experimental Therapeutics **348**(1), 116–124 (2013). doi:10.1124/jpet.113.208892

Table S1 Selection of questionable active compounds from the DRD2 dataset

Name	Structure	Comment
Tamoxifen	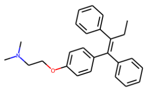	Selective estrogen receptor modulator
Disulfiram	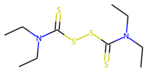	Covalent inhibitor of the acetaldehyde deshydrogenase, reactive disulfide bond
Amiodarone	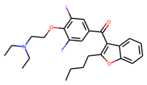	Anti-arrythmic drug, found active on H3 receptor but not to D2 [1]
Benzothiazyl Disulfide	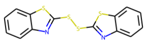	Contact allergen, features a reactive disulfide bond
Gentian Violet	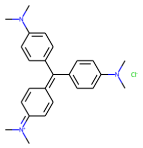	Dye, known toxic to the CHO cells used in the assay

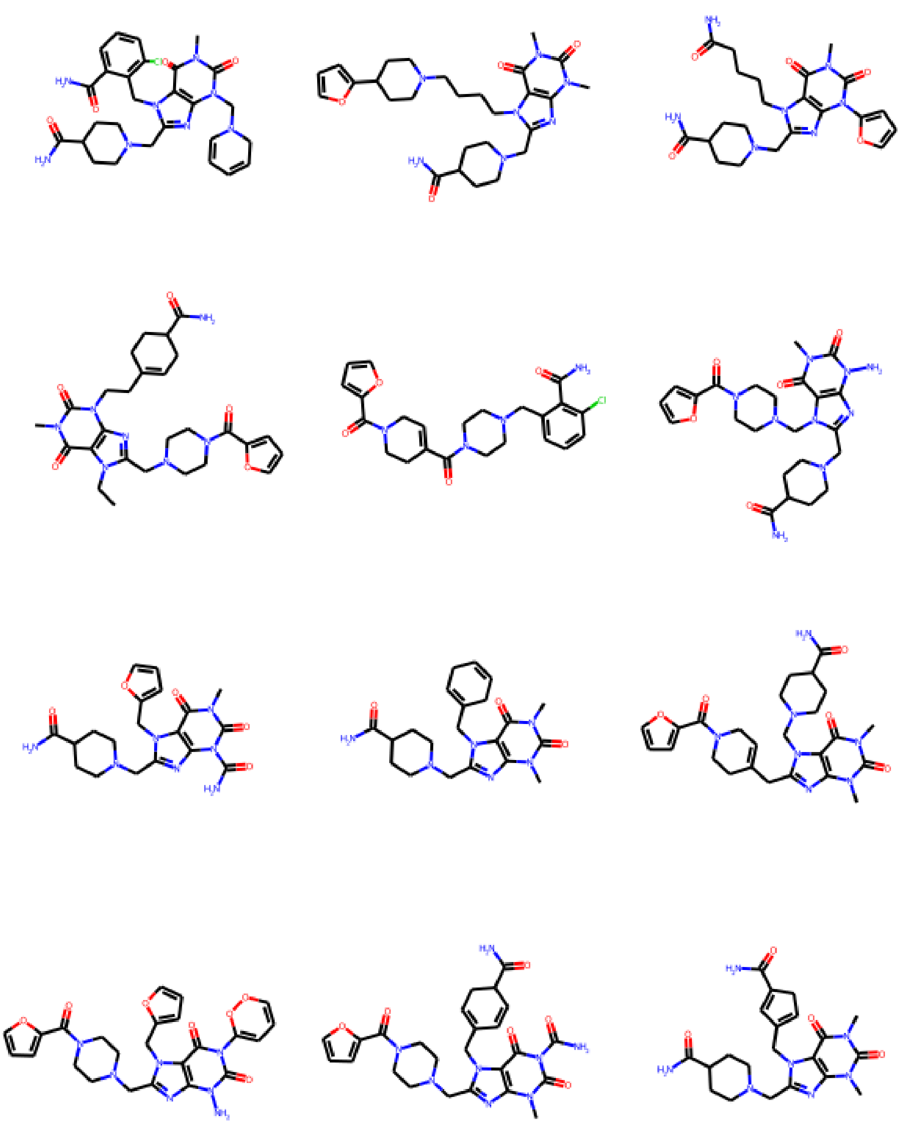

Figure S1 Random set of the final samples generated on the ALDH1 dataset with the graph genetic algorithm.

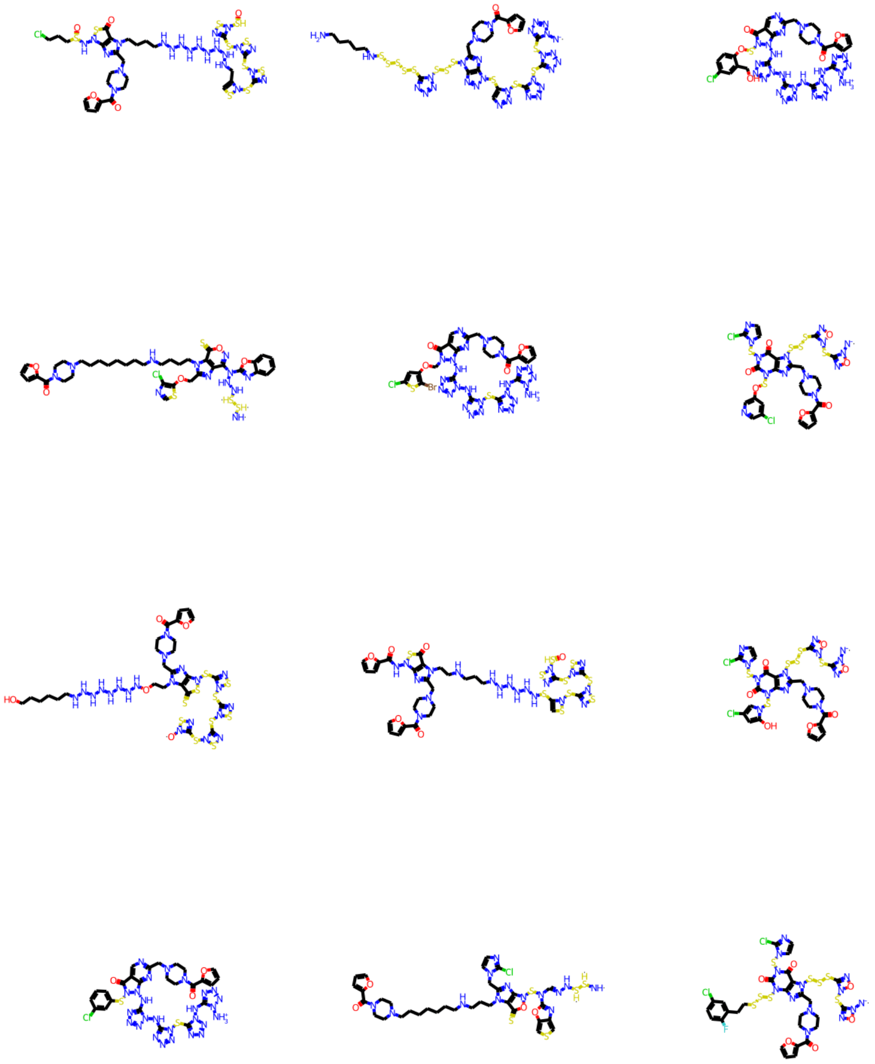

Figure S2 Random set of the final samples generated on the ALDH1 dataset with the SMILES-LSTM algorithm. Those chemical structures are highly non drug-like due to e.g. the repetition of tetrazole rings, long chains with sulphur-sulphur bonds and other heteroaromatic-heteroaromatic single bonds.

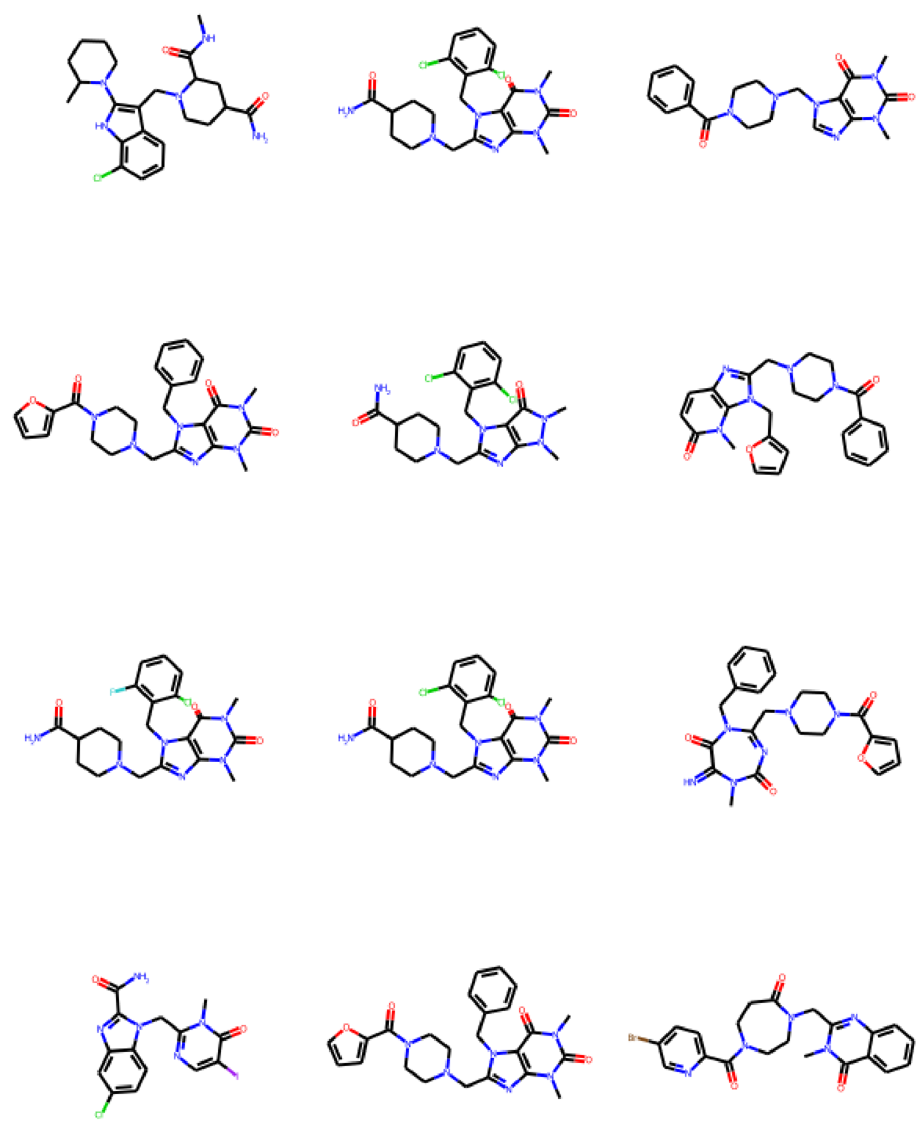

Figure S3 Random set of the final samples generated on the ALDH1 dataset with the MSO algorithm.

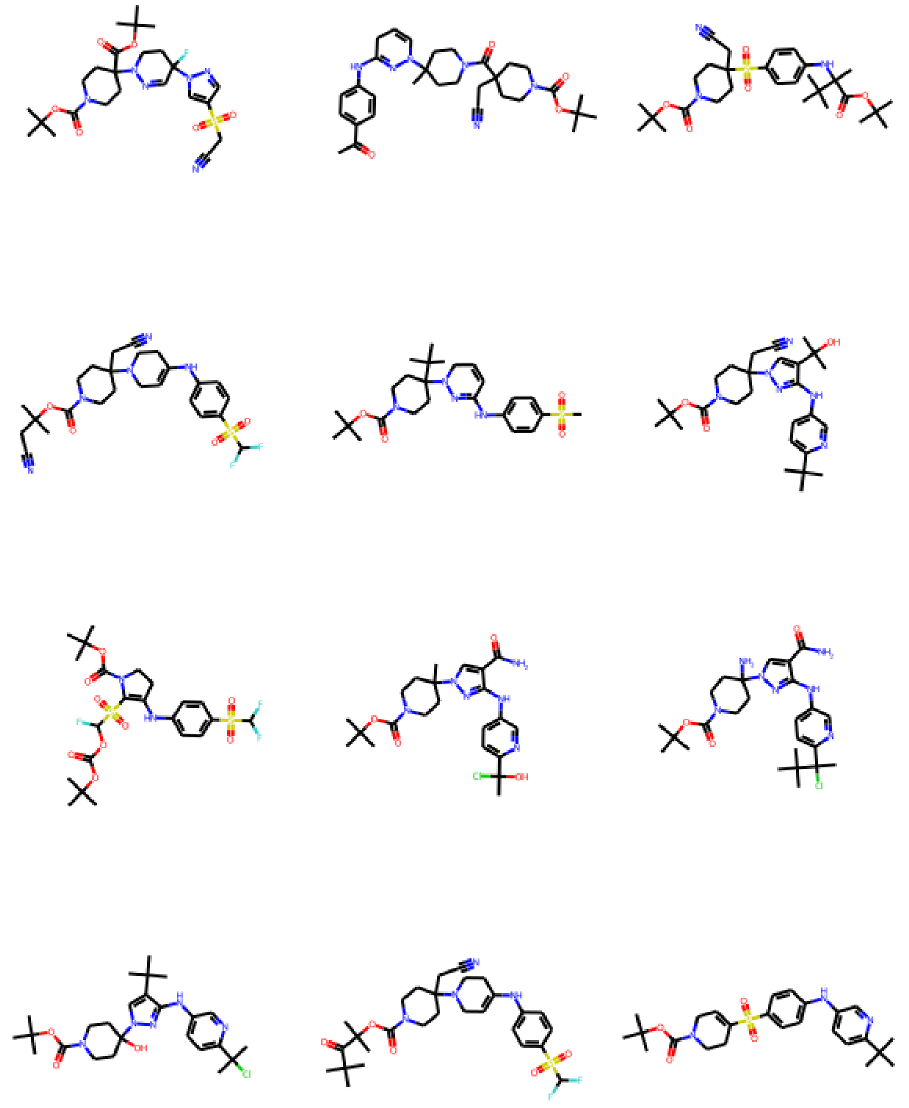

Figure S4 Random set of the final samples generated on the JAK2 (with modified architecture for the predictive model) dataset with the the graph genetic algorithm.

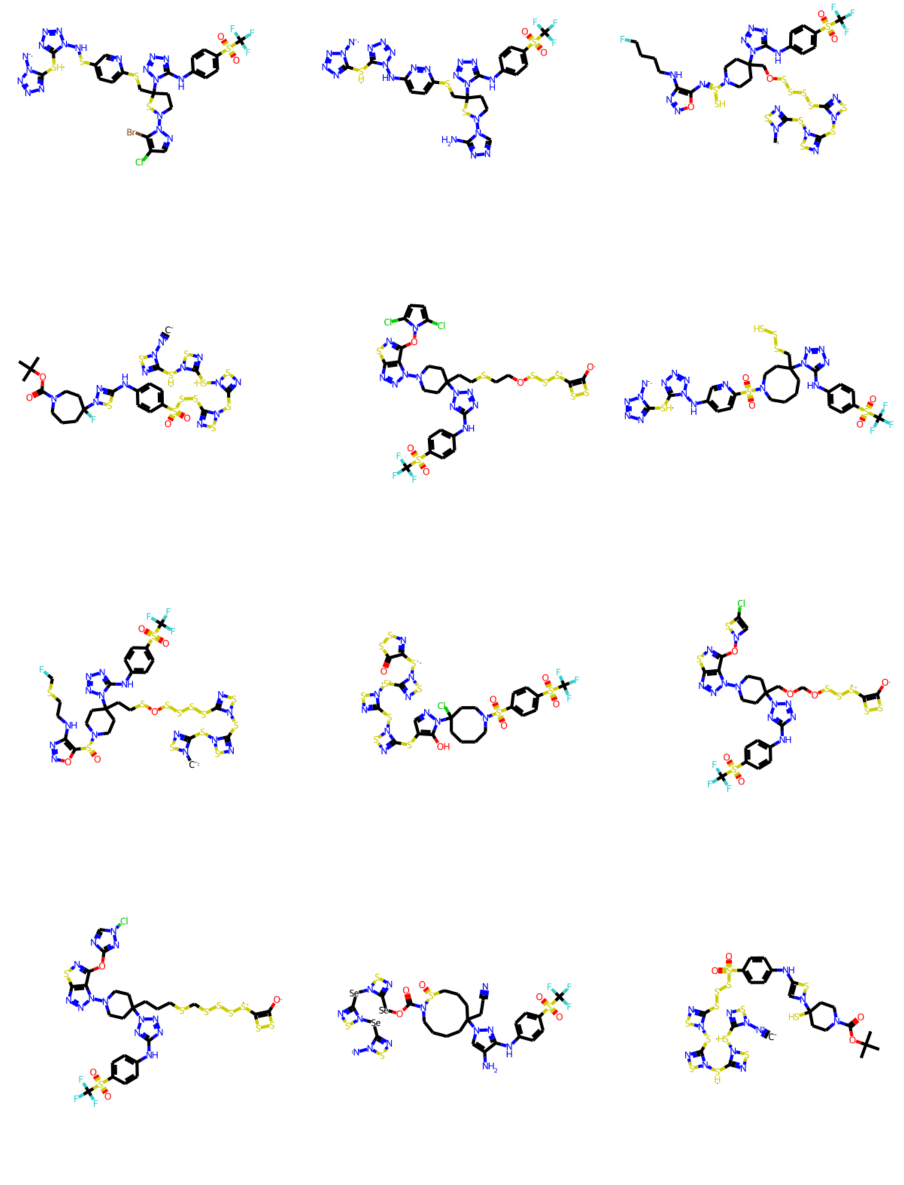

Figure S5 Random set of the final samples generated on the JAK2 (with modified architecture for the predictive model) dataset with the SMILES-LSTM algorithm. We observed similar non drug-like patterns as in Figure S2.

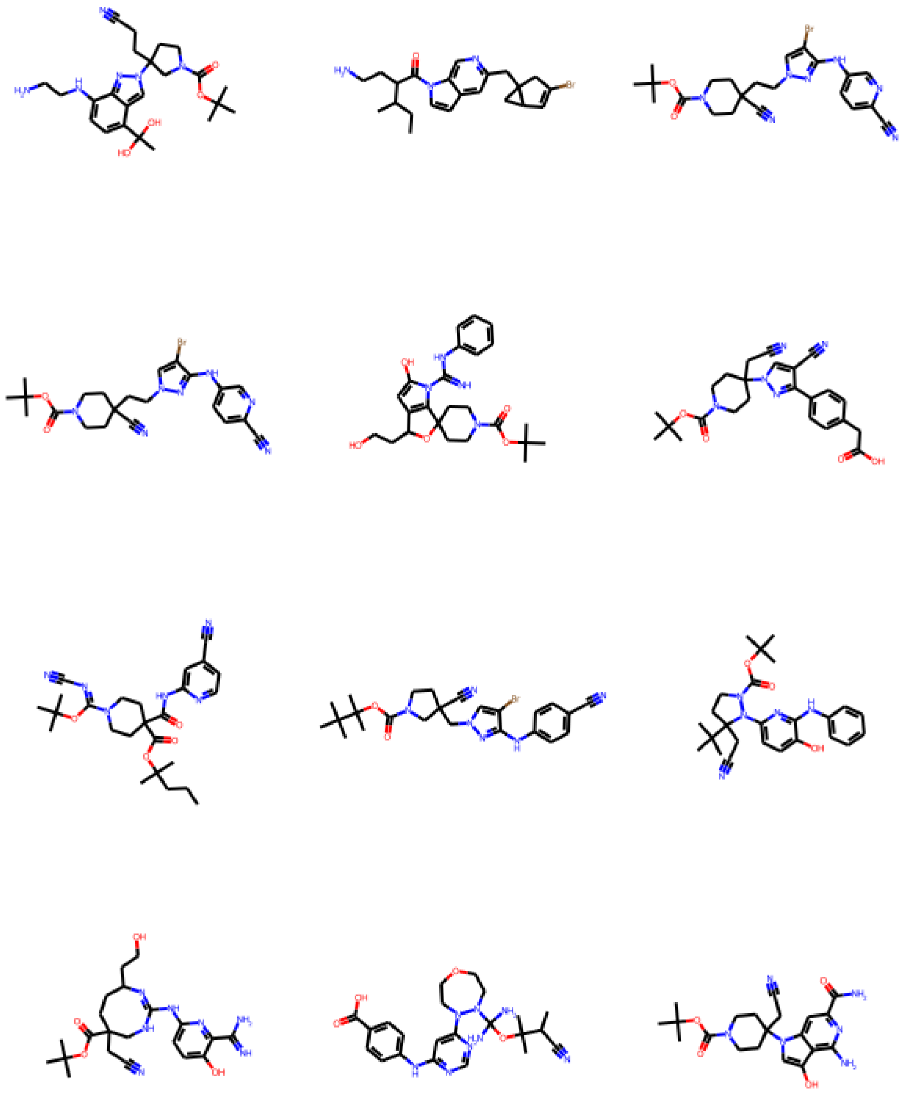

Figure S6 Random set of the final samples generated on the JAK2 (with modified architecture for the predictive model) dataset with the MSO algorithm.

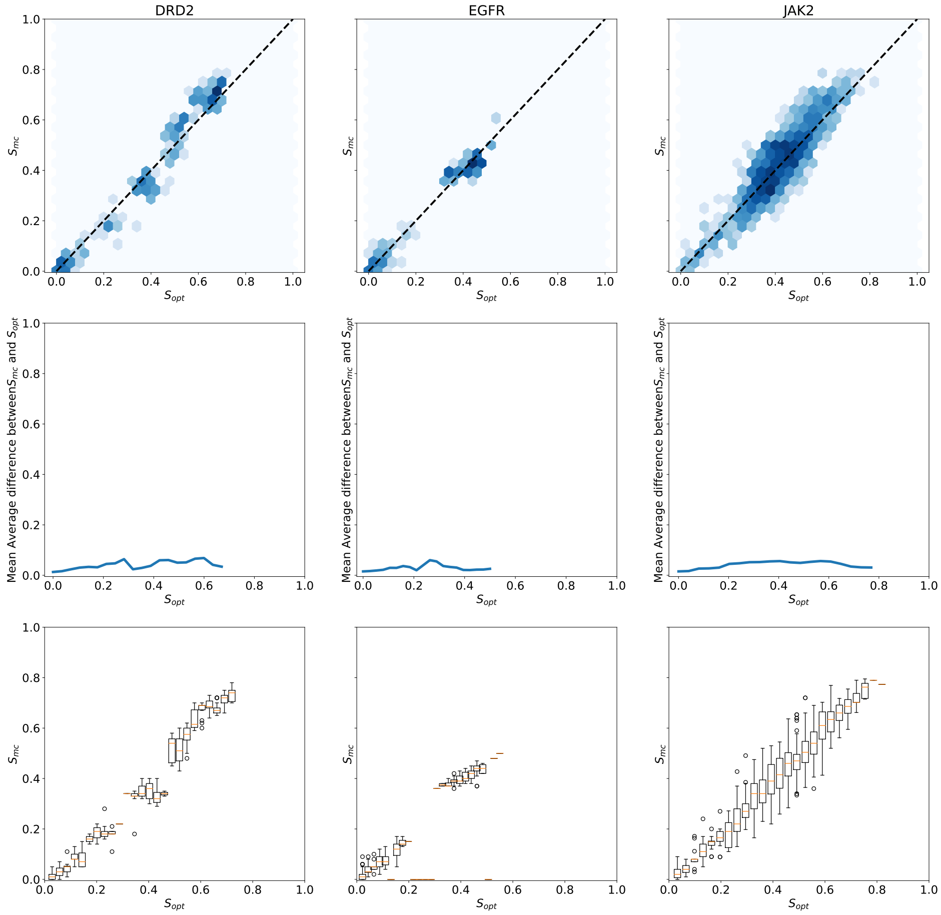

Figure S7 S_{opt} and S_{mc} in the DRD2, EGFR and JAK2 Topliss-augmented datasets. From top to bottom: hexbin plots (log scale) of data control as a function of optimization score; Mean Average Difference between S_{mc} and S_{opt} as a function of S_{opt} (at absciss x, the MAD plotted is the MAD for molecules with optimization scores higher than x); distribution of S_{mc} (95 CI) as a function of optimization score. For the second and third row, the lines and boxplots stop at absciss x_{max} for which there is no more samples with optimization scores higher than x_{max}.

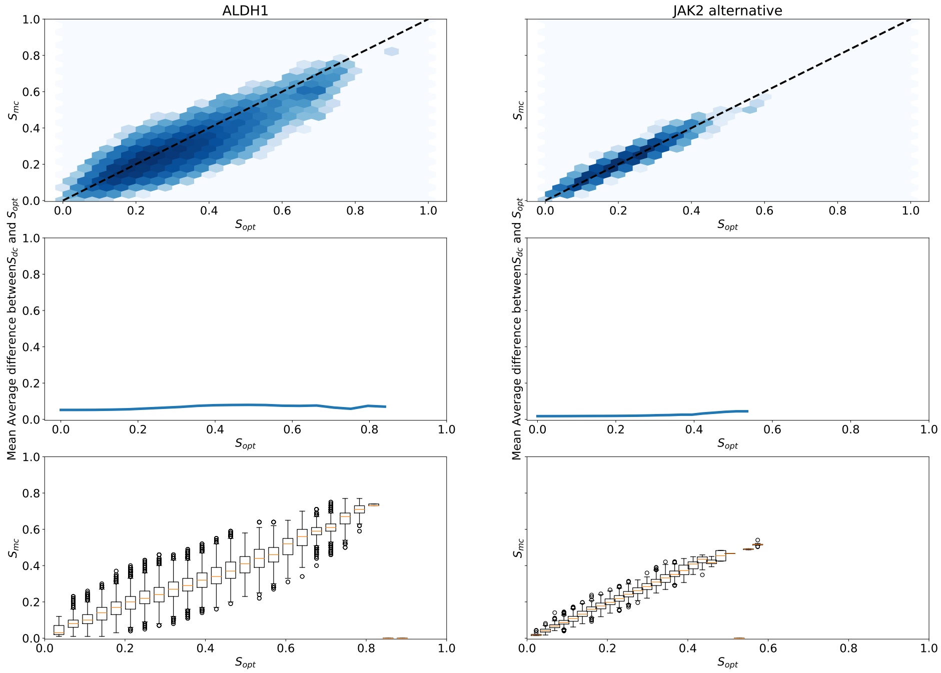

Figure S8 S_{opt} and S_{mc} in the ALDH1 and JAK2 modified Topliss-augmented datasets. From top to bottom: hexbin plots (log scale) of data control as a function of optimization score; Mean Average Difference between S_{mc} and S_{opt} as a function of S_{opt} (at absciss x, the MAD plotted is the MAD for molecules with optimization scores higher than x); distribution of S_{dc} (95 CI) as a function of optimization score. For the second and third row, the lines and boxplots stop at absciss x_{max} for which there is no more samples with optimization scores higher than x_{max}.

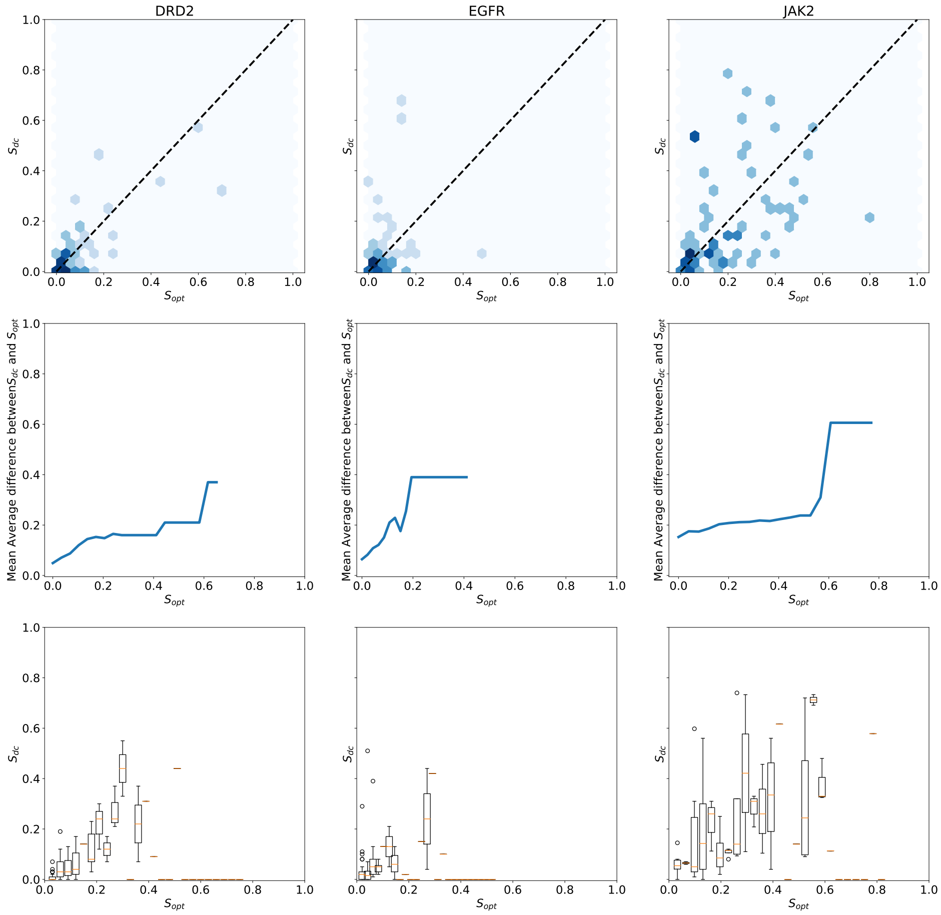

Figure S9 S_{opt} and S_{dc} in the DRD2, EGFR and JAK2 datasets without Topliss augmentation. The fact that only a few molecules have a high S_{opt} makes the evaluation of related quantities noisier than with data augmentation. From top to bottom: hexbin plots (log scale) of data control as a function of optimization score; Mean Average Difference between S_{dc} and S_{opt} as a function of S_{opt} (at absciss x, the MAD plotted is the MAD for molecules with optimization scores higher than x); distribution of S_{mc} (95 CI) as a function of optimization score. For the second and third row, the lines and boxplots stop at absciss x_{max} for which there is no more samples with optimization scores higher than x_{max}.

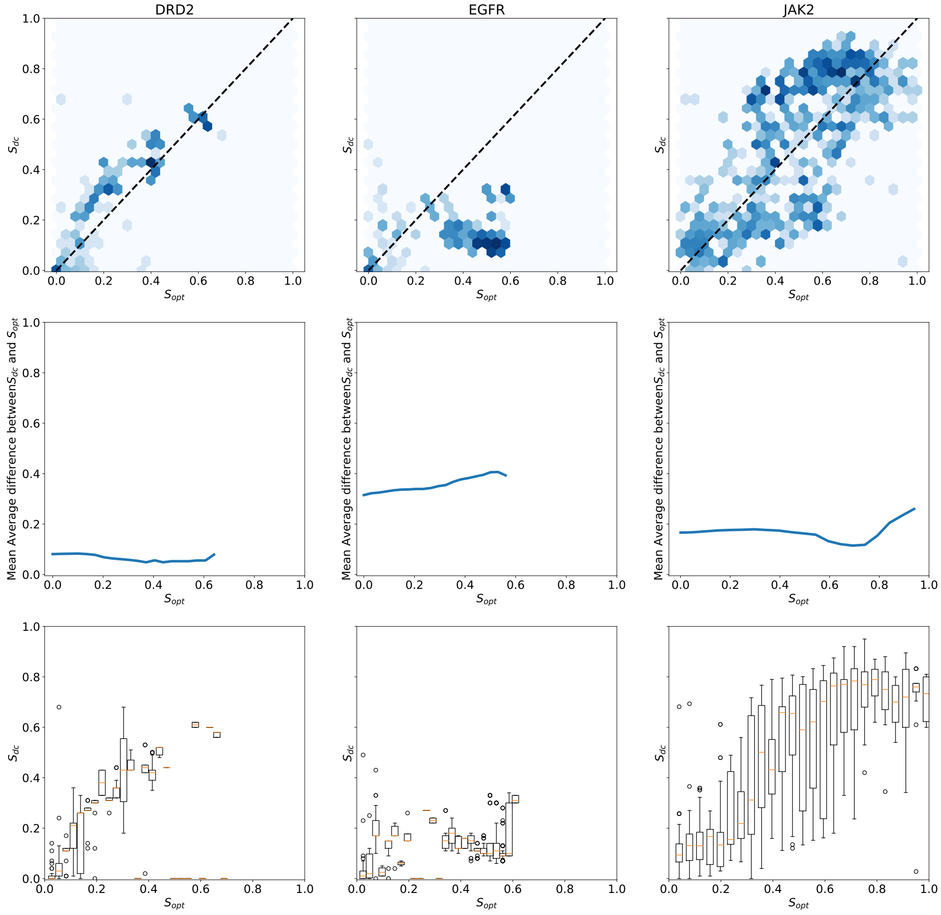

Figure S10 S_{opt} and S_{dc} in the DRD2 (ROC-AUC: 0.78, Average Precision: 0.2), EGFR (ROC-AUC:0.93, Average Precision: 0.67) and JAK2 (ROC-AUC:0.69, Average Precision: 0.41) Topliss-augmented datasets, when the classifiers use a combination of physico-chemical descriptors (number of hydrogen bonds donors and acceptors, number of rings, number of rotatable bonds, total polar surface area, Crippen descriptors (ClogP and molar refractivity), molecular weight, fraction of SP3 carbons, the ratio of atoms in the Murcko scaffold on the total number of heavy atoms, the number of heavy atoms, the maximum and minimum cycle size, the minimal, maximal and total charge and the number of chiral centers, as implemented in the RDKit). From top to bottom: hexbin plots (log scale) of data control as a function of optimization score; Mean Average Difference between S_{dc} and S_{opt} as a function of S_{opt} (at absciss x, the MAD plotted is the MAD for molecules with optimization scores higher than x); distribution of S_{mc} (95 CI) as a function of optimization score. For the second and third row, the lines and boxplots stop at absciss x_{max} for which there is no more samples with optimization scores higher than x_{max}.

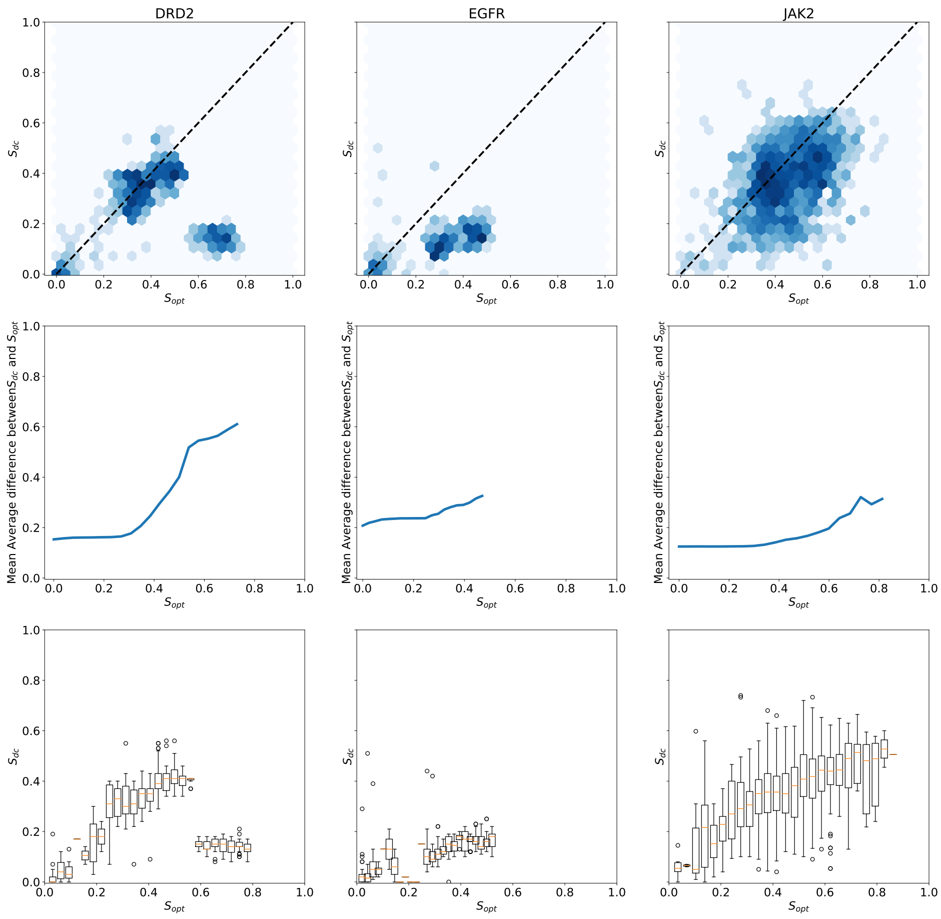

Figure S11 S_{opt} and S_{dc} in the DRD2 (ROC-AUC: 0.95, Average Precision: 0.56), EGFR (ROC-AUC:0.88, Average Precision: 0.24) and JAK2 (ROC-AUC:0.67, Average Precision: 0.37) Topliss-augmented datasets, when the classifiers use Atom-Pair descriptors (as implemented in the RDKit). From top to bottom: hexbin plots (log scale) of data control as a function of optimization score; Mean Average Difference between S_{dc} and S_{opt} as a function of S_{opt} (at absciss x, the MAD plotted is the MAD for molecules with optimization scores higher than x); distribution of S_{mc} (95 CI) as a function of optimization score. For the second and third row, the lines and boxplots stop at absciss x_{max} for which there is no more samples with optimization scores higher than x_{max}.
